# Selection for background matching drives sympatric speciation in Wall Gecko

**DOI:** 10.1038/s41598-018-37587-3

**Published:** 2019-02-04

**Authors:** Domenico Fulgione, Maria Buglione, Daniela Rippa, Martina Trapanese, Simona Petrelli, Daria Maria Monti, Massimo Aria, Rita Del Giudice, Valeria Maselli

**Affiliations:** 1 Department of Biology, University of Naples Federico II, Via Cupa Nuova Cinthia 26, 80126 Naples, Italy; 2 Department of Chemical Sciences, University of Naples Federico II, Via Cupa Nuova Cinthia 26, 80126 Naples, Italy; 3 Department of Economics and Statistics, University of Naples Federico II, Via Cupa Nuova Cinthia 26, 80126 Naples, Italy

**Keywords:** Speciation, Herpetology

## Abstract

The Wall Gecko shows heterogeneous colour pattern, which may vary among individuals, depending on the time of day and on the habitat segregation. Nocturnal pale geckos live exclusively on walls. Diurnal dark geckos preferentially live on olive tree trunks, demonstrating an ability to change skin colour that is superior to that of the pale gecko and allows diurnal geckos becoming camouflaged on the diverse substrates occupied during the day. In our study, the nocturnal/pale/wall and diurnal/dark/trunk geckos could be considered the extremes of an ecological cline of morphological variation on which divergent selection may be acting. Combining the effect of balancing selection on nocturnal geckos and disruptive selection between two sympatric populations could lead to speciation. All geckos analysed here belong to the same species, as confirmed by genetic characterization, however diurnal and nocturnal gecko populations seem to be in an early stage of incipient speciation. These two different morphs still combine genes, as revealed by neutral genetic markers, yet they show complete separation according to the analyses of mtDNA coding genes. Experimental results show that diurnal and nocturnal geckos do not swap their niches, likely because the predation pressure causes severe selection for background matching. Genomic analysis of complete mtDNA suggests that nocturnal geckos seem to be under balancing selection perhaps due to the narrow niche in which they live, whereas the daytime population has more opportunity in fitting into the multiple available niches, and they experience positive selection. Here we hypothesize that the ecological segregation that we are witnessing between the nocturnal and diurnal geckos, can lead to a ecological speciation.

## Introduction

Divergent natural selection caused by shifts in ecology or invasions of novel habitats plays an important role in adaptive population divergence and speciation^1^. Selection is ecological when it arises from the interaction of individuals with their environment during resource acquisition^2^, it can be divergent when it acts in contrasting directions on different populations. This includes the special case in which selection favours opposite, usually extreme, phenotypes within a single population (i.e. disruptive selection). The latter is the dominant form of selection in theoretical models of sympatric speciation driven by natural selection^3,4^.

Speciation resulting from ecologically-based divergent selection has recently received considerable attention^2,5^, although only a few strong cases have been reported for this phenomenon, involving arthropods^6^, fishes^7^ and whales^8^. For terrestrial vertebrates, Darwin’s finches^9–11^ and finches on Tristan da Cunha^12,13^ have been described, in which a typical example of adaptive radiation acting in parallel with ecological speciation has been observed.

**Figure 1:**
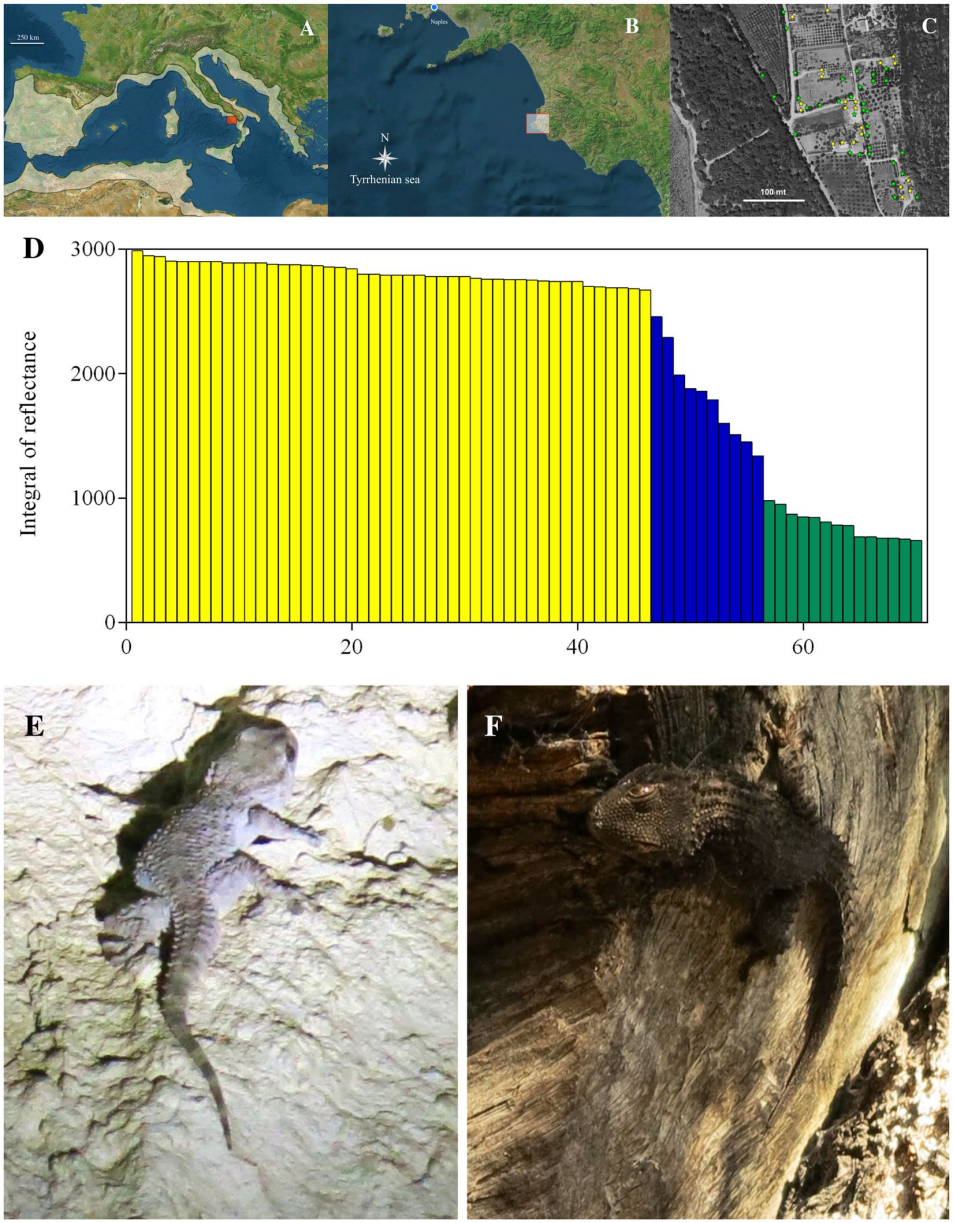
Range, study area and identification of gecko morphoypes. (**A**) General geographic range of Wall Gecko, *Tarentola mauritanica* and (**B**) the study area in the box (Punta Licosa geographical coordinates 40°15′N, 14°54′E) located in a remote area in the Cilento, Vallo di Diano and Alburni National Park (South Italy) considered as a landscape island, far from maritime or other commercial activities, with very low human incidence. (**C**) The sampling sites of the nocturnal (yellow stars) and diurnal (green stars) geckos in the study area show they live in sympatry. (**D**) Spectrophotometric analysis of 70 geckos’ body colouration measured with the integrals subtend the reflectance curve measured in six positions of dorsal skin; the slope of the linear function identifies the breakpoint, clearly distinguishing among pale (yellow), intermediate (blue) and dark (green) samples. Different geckos’ morphotypes in their natural habitat, pale gecko on lighted wall (**E**) and dark gecko on olive trunk tree (**F**). Satellite images in Fig. 1A–C were obtained from satellite image attributed to Image ©2018 Google, Data SIO, NOAA, U.S. Navy, NGA, GEBCO, TerraMetrics, Map data ©2018 Google Italia, by Domenico Fulgione.

The Wall Gecko (*Tarentola mauritanica*) is a common medium size lizard, widely distributed in the western Mediterranean basin (Fig. 1A). The Wall Gecko has specialised microscopic suction pads on its toes, known as ‘*setae*’, which allow the gecko to climb flat surfaces including walls and ceilings^14,15^.

Several specialist and enthusiast authors have shown, in local atlas, reports and web movies, the occurrence of an extraordinary interspecific colour variability^16^. Some reports describe animals with heterogeneous colour pattern ranging from whitish grey to brownish-black, with darker and lighter spots^17,18^, which may vary among individuals, depending on the time of day and the physiological conditions^19^. Geckos are described as primarily nocturnal, with some basking during the day to regulate their body temperature^20–23^; these occupy a more limited diurnal living space than the purely nocturnal type^24^. They are typically associated with vertical surfaces^21^ and frequently use areas around artificial light sources for foraging^25^. Mating occurs between March and July with a second mating sometimes taking place in autumn^26,27^.

Based on these reports and original observations, our group has demonstrated that the Wall Gecko has the ability to change the colour of its skin to better match its surroundings and make it less conspicuous to predators^28–30^. In addition to this individual colour variation, the species shows a gradient of ranging from dark geckos, living by day, to pale geckos that meet at night.

Here we hypothesize that this diversity could induce segregation and drive a population division mainly based on a temporal split (day - night). Moreover, in our field observations we have noted mating events both at night, between the pale gecko, and during the day between dark geckos.

Thus, we have performed an ecological survey and several experiments, all aimed at identifying the ultimate and proximate causes of morphological and ecological variation in the Wall Gecko, and its proximate and remote causality. In particular, we have utilized a broad and multidisciplinary experimental approach, which is summarized in Fig. S1 (graphical abstract).

We worked in a remote study area of the Cilento Vallo di Diano and Alburni National Park (South Italy), considered a habitat island (*sensu* Whittaker and Fernandez-Palacios 2007^31^), far from maritime or other commercial activities, with a very low human incidence. This is to avoid the introduction of non-native geckos to the area being investigated. In fact, dispersion of gekkonids within their range has been altered through passive transport by man, mainly at trading harbours.

Our data describe, for the first time, evidence of incipient ecological speciation in sympatric populations of the Wall Gecko. Firstly, we quantify colour variation by measuring the reflectance of the skin of a small isolate population, grouping it into three classes: pale, dark and intermediate geckos. The two extreme colour phenotypes are separated temporally. To investigate the split in the two ecological niches, we have designed experiments of population genetics and capture/marking/release to determine how much they are currently separated. The amount of melanin in the skin determines the ability to change skin colour, but only so far as to better adapt to the niche, not to shift into the other niche. We provide support that predation affects segregation of diurnal dark and nocturnal pale geckos, with effects also on the genetic variability.

## Methods

### Study area and sampling

The study was conducted in Punta Licosa (40°15′N, 14°54′E), located in Southern Italy, near the coast of the province of Salerno, characterized by Mediterranean vegetation, olive tree cultivation, rural buildings and stone walls (Fig. 1A–C).

We caught 70 Wall Geckos by hand or by noose. After experimentation, 50 geckos were released at the point of capture and 20 individuals were sacrificed for sampling tissue.

### Gecko presence and distribution

The study area was visited during the day and night to assess the presence/absence of geckos. We examined the external surfaces of each structure (walls, olive tree trunks, verandas, other human constructions, etc.) using binoculars (Swarovski 10 × 40) and torches (Ledlenser MT14), annotating the characteristics, the number, the position and phenotypic traits of those observed. Each site was surveyed for an average of 15 minutes, either from 7.00 am to 6.30 pm or 7.00 pm to 6.30 am, for diurnal and nocturnal observations, respectively. Surveys were not conducted during rains or when the temperature was below 18 °C.

### Predation pressure assay

Predation pressure on geckos was tested using plasticine models^7,32–37^. We produced 40 realistic models for each form of adult gecko, constructed by pouring non-toxic plasticine into a flexible mould using a preserved museum specimen and then painting to resemble the colours of wild pale and dark animals. Each plasticine coloured model population was divided in two sub-populations put on trees and walls in the study area (N = 20 and N = 20, respectively) approximately in the same position where a real gecko was previously observed.

After leaving pale and dark models on the wall of houses during night, and leaving pale and dark models on the tree trunks during day, we recorded the type and frequency of beak marks by stereomicroscope LeicaEZ40 (Leica Microsystems srl, Milan, Italy). We considered a model as “attacked” when it exhibited at least one clear beak mark or when it had disappeared. The predation signs were assigned to the most likely predator, based on morphological features. We divided predators in “aerial” (mostly birds as *Strix aluco, Athene noctua, Falco tinnunculus*, *Lanius collurio* and *Corvus corone*) and “terrestrial” (mostly small mammals as *Rattus sp*.). These types of predators are highly dependent upon vision to detect their prey and to perform the accurate orienting and approach behaviours leading to capture small animals^38–40^. Then, we compared the frequency of predation event for pale models put on the tree trunks (during the day) and on walls (during the night), and the same it was made for dark models. The significance of the data from this analysis was evaluated by a chi-square test.

### Reflectance skin assay

The animals’ body colouration was objectively determined measuring the skin reflectance by spectrophotometry on 70 individuals as reported^29,41^.

Reflectance percentage (R%) was assayed among each individual. The spectral range was considered between 300 and 700 nm^30^. The average of the integrals subtended the reflectance curve within the range of wavelength of 300 and 700 nm was assumed to be representative of the whole back^29,41,42^.

The differences between morphotypes are identified as breakpoints of clinal variation with *Strucchange* approach^43^.

In the present study, we investigated also if geckos assigned to two extreme phenotype populations (diurnal dark and nocturnal pale) have differential ability to modulate their darkness (see^29,30^), comparing their range of skin colour variability. This ability was estimated by measuring skin reflectance after placing the gecko (dark N = 14 or pale N = 15) for 3 hours in a white box and after in a black box (for 3 hours). The difference in the skin colour is outlined as a curve of mean reflectance spectra (%) in which the mode points out in the visible colour. The variation around each curve represents the reflectance expressed under the experimental conditions described above. Values, which extends above the curve express change of the skin toward pale colour, while those under express the change towards dark colour. The greater variation range is indicative of a greater ability to change one’s skin colour.

We compared firstly the observed colour (integral under reflectance curve) in the two populations by a two-way ANOVA analysis in XLSTAT software^44^. In this analysis, the first way of variation was considered the base colour, and the second way of variation was considered the ability to modulate darkness after experimental condition (in white and black boxes).

### Population genomic of mtDNA

Due to the smaller effective population size of mitochondrial DNA, haplotypes frequencies could diverge more quickly and result in larger values of differentiation measures^45^. There is a great deal to be learned about how selection shapes mitochondrial genome evolution, nevertheless mitochondrially encoded proteins evolve much more slowly than predicted by the high mutation rate in the mitochondrial genome^46^, so purifying selection on nonsynonymous mutations is clearly an important component of mitochondrial evolution^47^. Coding regions of mitogenome contain more candidate sites for selection and of functional impact suggesting that its variations can related to protein function^48–50^. More specifically, this markers by targeting genetic regions that are directly influenced by natural selection, suggesting that local adaptation is correlated to the genetic diversity and differentiation of populations^51^.

Total mitochondrial DNA (mtDNA) was extracted from tail tissue of diurnal/dark geckos collected on the trunks (N = 9) and nocturnal/pale geckos collected on the wall (N = 11), using plasmid miniprep PureLink HiPure Plasmid DNA Purification Kits (Invitrogen), according to manufacturer’s recommendations, except for using 25 mg of tissue and initial mechanical disruption of samples for 1 h in a PBS buffer. Blank extractions without samples were systematically performed to monitor potential cross-contaminations.

Before sequencing, we checked concentration and quality of DNA using the Qubit Fluorometer (Thermofisher scientific) and the Agilent 4200 TapeStation system (Agilent Technologies), respectively.

Next-generation sequencing (NGS) of the total mitochondrial genome was performed on the Illumina 2500 platform using the Nextera XT DNA Sample Preparation protocol, according to manufacturer’s recommendations, at the Genomix4Life Srl.

FastQC software v0.11.4^52^ was used to quality control raw sequence data. The Trimmomatic software v0.35^53^, in paired end mode, was implemented to trim and crop the low quality raw reads (Q < 28), and to clip the primers and Illumina adapters.

For assembly, we used the software Geneious version R9.1^54^, in mapping assembler mode. For each sample, we performed a *de novo* assembly and the filtered reads (forward and reverse) were used to create overlapping and contiguous sequences (contigs). To check the quality of assembly, the contig sequences were mapping against a *Tarentola mauritanica* reference sequence [Accession number EU443255].

We used DnaSP^55^ to calculate nucleotide diversity (π) and haplotype diversity (Hd), to compute *F*_*ST*_ for coding region in mtDNA and grouped individuals according to the ecological segregation (nocturnal/dark/wall gecko and diurnal/pale/trunk gecko)^56^.

We used DnaSP also to perform Tajima’s D^57^, meanwhile Z-test^58^ (p-values were generated using 1000 simulations) and statistic test of normalized dN-dS were performed in Mega 7.0^59^. The statistic test of normalized dN-dS was used for detecting codons that having undergone positive selection, where dS is the number of synonymous substitution per site and dN is the number of non-synonymous per site. A positive value for the test statistic indicates an overabundance of non-synonymous substitution.

Furthermore, to evaluate differences in evolutionary rates and to highlight differences between dN/dS in diurnal and nocturnal populations, we used permutation tests^60^, performed in R cran^61^ with 100,000 permutation, and with the script for running permutation tests described by Schrader and co-workers^60^.

### Phylogenetic characterization

mtDNA sequences were aligned using software Geneious version R9.1 (http://www.geneious.com)^54^, with 21 specimens representative of three different lineages of *T. mauritanica:* European (DB11105, DB9107, DB9112, DB9113, DB9115, and DB9116); Iberian Peninsula (DB3832, DB3839, DB3843, DB3846, DB3853), Central and Southern Morocco (DB11003, DB11004, DB11007, DB11008, DB11009, DB11013, DB11022, DB11035, DB11042, DB2635)^62^ and *Gekko gecko* was designated as the outgroup (AY282753). Calculation of the likelihood scores and choice of the best model of sequence evolution was carried out on whole mtDNA genome (e.g. protein coding, rRNA, tRNA genes), using jModeltest v2.1.4^63^, under the Akaike Information Criterion^64^, selecting the TIM1 + G model. A Bayesian analysis was performed using Mr.Bayes v.3.1.2^65,66^. The Bayesian posterior probabilities were estimated using a Metropolis-coupled, Markov chain Monte Carlo (MC–MCMC) sampling approach. Both runs started with random starting trees and ran for 2 × 10^6^ generations, saving one tree in each 100 generations. Using the software Tracer v1.4^67^ it was confirmed that all parameters had an ESS > 100 after burnin. The remaining trees were combined and a 50% majority consensus tree was generated.

### Population genetics of neutral loci

To assess genetic population structure, we collected 63 tips of tail from geckos belonging to nocturnal/wall (N = 32), diurnal/trunk populations (N = 13) and diurnal no-trunk population (N = 18).

We analysed 8 polymorphic microsatellite loci: 6 (Tb8, Tb35, Tb71, Tb192, Tb213, Tb240) developed from *Tarentola boettgeri*^68,69^ and 2 (Mt6 and Mt27) developed from *Tarentola mauritanica*^62^. Polymerase chain reaction (PCR) amplifications were carried out in 10 μl final volumes containing 20 ng of genomic DNA, 0.50 μM of each primer, 10X PCR buffer (160 mM (NH4)_2_SO_4_; 670 mM Tris-HCl pH 8.8; 15 mM MgCl_2_; 0.1% Tween 20), 0.2 U Taq polymerase (SIGMA Dream Taq), 0.25 mM each dNTP. The thermocycler profile started with an initial denaturation step at 94 °C for 3 min, followed by 35 cycles at 94 °C for 30 s, temperature annealing at 50–55 °C for 1 min, 72 °C for 1 min followed by 72 °C for 5 min. A negative control was run with each round of PCR.

Polymorphisms of microsatellite were determined using forward primers, end-labelled with a fluorescent dye group (FAM or HEX, MWG Biotech). An internal size standard (LIZ500) was run with our samples. Amplified DNA fragments were electrophoresed using an ABI 3100 automated sequencing instrument (Perkin-Elmer) sequencer, and their genotypes were analysed with GeneMarker Software, version 4.0.

Microsatellite genotypes and sample spatial location data were analysed for the 8 loci in GENELAND package in R^70^. K was set at a minimum of 1 and a maximum of 6. An allele frequency uncorrelated model was set, with 1,000,000 Markov Chain Monte Carlo (MCMC) iterations and thinning of 10, and null allele frequencies were explicitly considered.

To identify the population affinities of individual samples, we used a Bayesian clustering method implemented in STRUCTURE 5^71,72^, running using the following parameters: admixture model of ancestry, correlated allele frequency model, a burn-in period of 10,000 simulations followed by a run length of 2,000,000 MCMC simulations. K, ranging from 1 to 5, was tested in three independent runs to establish consistency and the probability of data (Ln) was estimated in each without using any prior population information, so that each individual was assigned to a cluster based upon its multilocus genotype profile.

Molecular variance among individuals within and between phenotype groups was visualized in three-dimensional factorial correspondence analysis implemented in GENETIX 4.05^73^.

Global population differentiation was estimated using Wright’s *Fst*^74^, and pairwise *Fst* values were estimated using *θ*^75^ in GENETIX 4.05. *Fst* p-values were computed using a permutation approach (2,000 iterations) as implemented in GENETIX 4.05.

### Melanin and Melanocyte Stimulating Hormone (α-MSH) assay

Skin and plasma were collected from 5 nocturnal/pale/wall geckos and 5 diurnal/dark/trunk geckos.

Melanin quantification was performed according to Kalie and co-authors^76^, except for tissues collection in 50 mM Tris-HCl pH 7.4, 300 mM NaCl, 0.5% NP-40 and protease inhibitors (Roche) and lysing of cells by freeze- thawing (3 cycles of dried ice-37 °C), Dounce homogenization (200 strokes) and sonication (2 min, 10 sec on and 10 sec off).

α-MSH levels in geckos were determined by the ELISA assay as described in Monti and co-authors^77^. The between-group (day *vs* night) difference was assessed by Student’s t test. Statistical significance was defined as p < 0.05.

### Marking, release and observation

We marked 50 pale geckos captured during one night on the walls of 15 houses, located in an area of approximatively 0.082 km^2^, and separated from to each other at least 30 meters. To facilitate visual recognition at a distance, a small amount of quick-drying nontoxic paints was applied with a brush^78–81^ on the back of each gecko. Then, we released the marked geckos at the point of capture on the walls of houses. During a second experimental section, we performed the same test on 50 dark geckos captured during one day on the trunks of about 50 olive trees located in an area of approximatively 0.087 km^2^ and separated from to each other at least 2 meters. Also in this case, we marked geckos and released them at the capture site. We performed a visual census during two days and two nights after the release, recording the number of marked geckos detected on wall of the houses and neighbouring olive tree trunks.

We observed the geckos by binoculars in daylight and hand-held torch during the night.

### Ethical statements

The animals were collected with the permissions of the county authorities (Cilento, Vallo di Diano and Alburni National Park prot. 0010678/2013). Animals were kept according to ministerial authorization (prot. 165/2006). All experimental protocols were approved by the Ethical Committee for Animal Experiments University of Naples Federico II (ID: 2013/0096988). All methods were carried out in accordance with relevant guidelines and regulations, according to Italian law (DL 26/2014).

## Results

**Figure 2:**
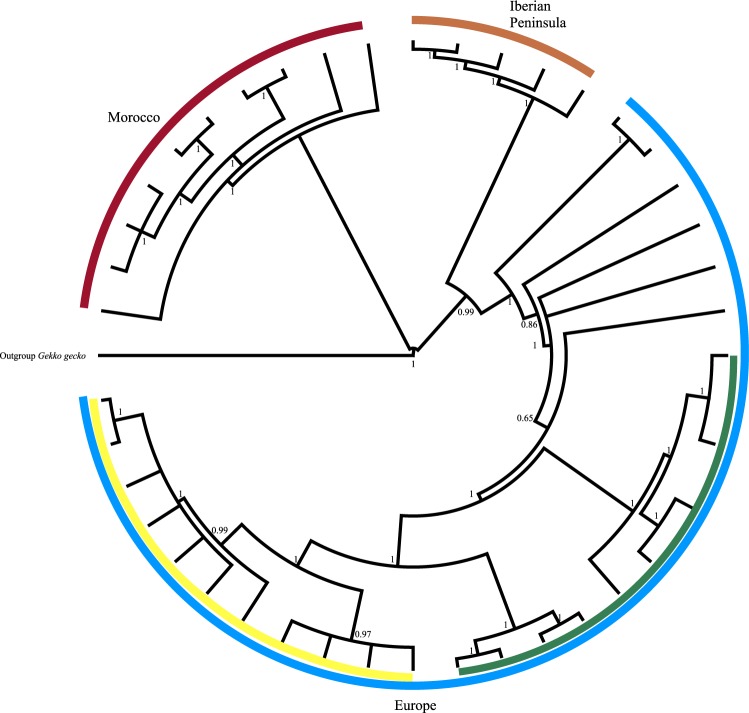
Bayesian trees of haplotypes of Palaearctic Geckos. mtDNA phylogenetic tree calculated for 15676 bp performed with TIM1 + G model. Bayesian posterior probabilities are represented over key nodes; The colours of each lineage are referred to the geographic distribution of the specimens used, Morocco (red), Iberian peninsula (orange), and Europe (blue) and within the European clade dark/diurnal (green) and pale/nocturnal (yellow) geckos.

Phylogenetic tree based on mtDNA (15,676 bp) shows that all geckos considered in our analysis belong to the same species and same biogeographical population (Fig. 2).

**Figure 3:**
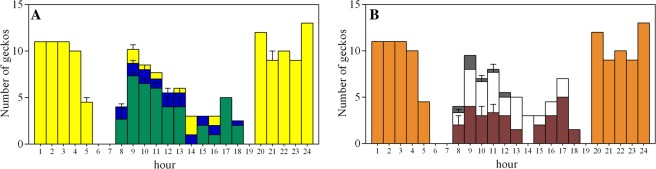
Visual census of the geckos’ population in Punta Licosa. (**A**) Mean of numbers of geckos of each morph recorded on 24 hours: dark/diurnal geckos (green), pale/nocturnal geckos (yellow), intermediate geckos (blue). (**B**) Mean of numbers of geckos recorded in 24 hours on different substrates: olive tree (brown), stone wall (white), house (orange), and other substrates (grey).

To perceive intraspecific colour variation, we measured the reflectance levels of 70 animals. Geckos followed a trend characterized by a strong clinal variation interposed between pale and dark individuals (Fig. 1D). The division into groups was done following an approach that evaluates the significant differences between the slopes of the Zeileis linear function^82^ and identifying the so-called breakpoints. We distinguished three morphotypes: pale (P, geckos with high reflectance, yellow bars in Fig. 1D), dark (D, characterized by low reflectance, green bars in Fig. 1D) and intermediate (I, grouping different geckos in a clinal variation, blue bars in Fig. 1D). Considering only the two homogeneous groups, according to the slope of the linear function, we identified breakpoints, with *Strucchange* approach^43^, between pale and dark morphotypes (Fig. 1E, F). Interestingly, a strong temporal split was observed also between nocturnal pale and diurnal dark geckos. Furthermore, only a small number of intermediate individuals were detected during the day (Fig. 3A). These observations are strictly linked to the backgrounds on which the geckos live. Indeed, during the night, pale animals were detected only on house walls, whereas during the day, dark geckos generally prefer the trunks of olive trees (Fig. 3B).

Statistical analysis on 255 observed geckos, aimed at testing the association between background substrate (wall, trunk and others) and phenotype (pale, dark and intermediate), confirmed that the habitat has significant effect on colour (Tables S1 and S2): 39.7% of the total nocturnal/pale geckos were found on (illuminated) house walls as background, whereas the diurnal/dark population prefers olive tree trunks (23.8%). Consequently, from diurnal observations, we found 13.6% of intermediate geckos on stone walls or other substrates. We do not exclude the fact that diurnal populations are segregating in more than one spatial niche, but this topic is outside the scope of this particular contribution and could be considered in future research.

**Figure 4:**
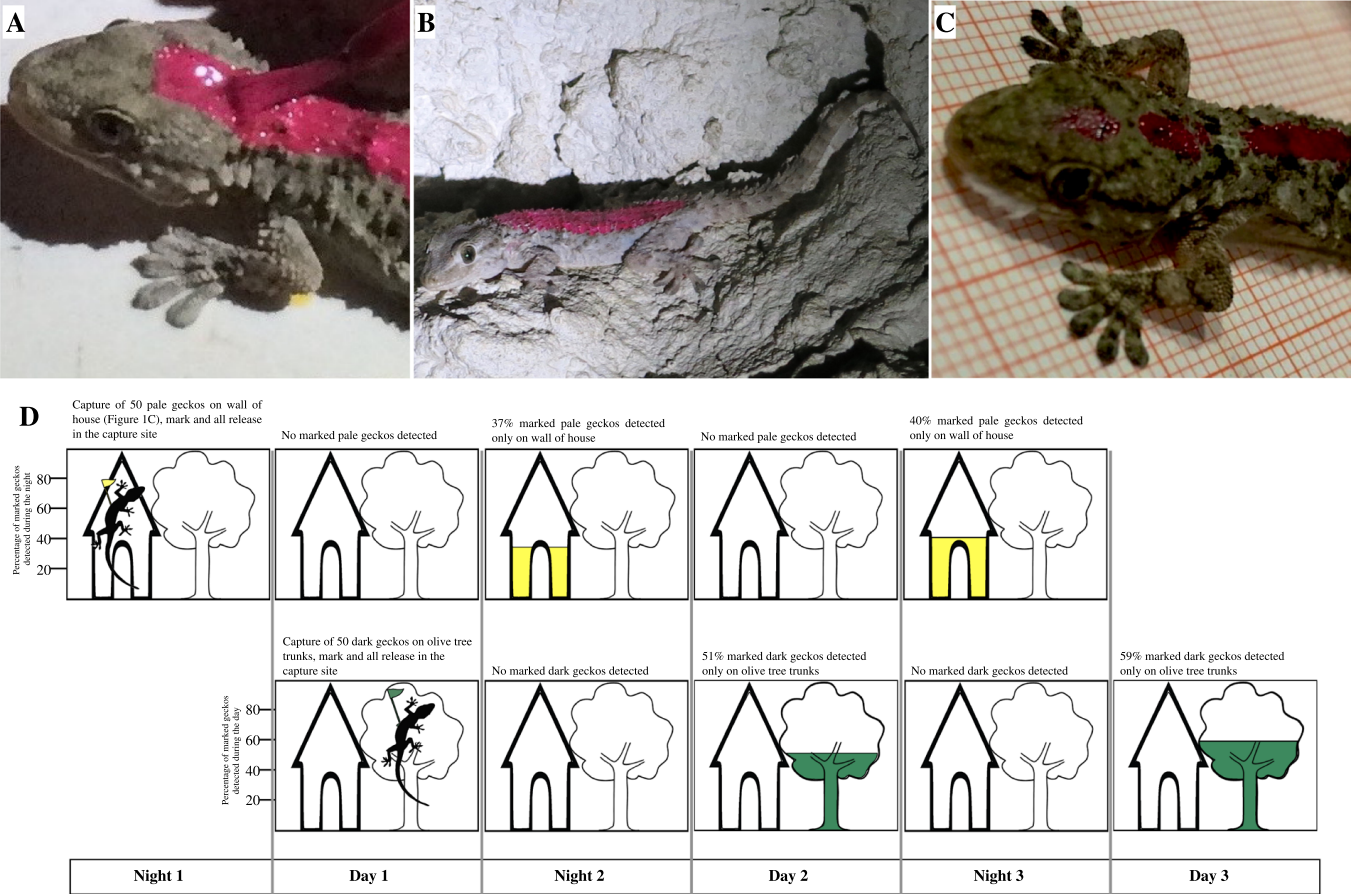
Geckos’ spatial/temporal segregation. (**A**, **B and C**) Marked geckos for recognition at distance in diurnal/nocturnal segregation analysis. (**D**) During one night 50 pale geckos were captured on a wall house (see dots in Fig. 1C), marked and they were all released on site of capture. The days after release (day 1 and day 2), no gecko was detected either on trunks and walls throughout the monitoring area highlighted in Fig. 1C. In the first night after the release, the number of marked geckos recorded near the release site was 37%, all on house walls (yellow). The second night after the release, 40% of marked geckos were recorded, again all on walls of house. The same experiment was conducted for dark gekos captured during day 1 on trunks (green). This was detected only on trunks in day 2 and 3.

To determine the habitat fidelity of the two populations, we set up the following experiment: 50 pale geckos were marked (Figs 4A, B, see also 1D) and released in order to discover their movements during the two following days (see supplementary information). According to capture-recapture model^83^, the marked geckos are mixed in the whole population and part of them will be detected in subsequent samplings. None of the marked geckos, released in the site of capture, were recorded during day time, neither on olive trees nor on house walls (Fig. 4C). Indeed, about 40% of pale marked geckos were found exclusively on walls of houses at the site of release, or close to it, during the first and second night after their release (Fig. 4D). The habitat fidelity was confirmed by the second capture-recapture experiment performed on dark diurnal geckos (Fig. 4C, see also Fig. 4D), found only on olive tree trunks (51% on the first day and 59% on the second day).

### Physiological status

**Figure 5:**
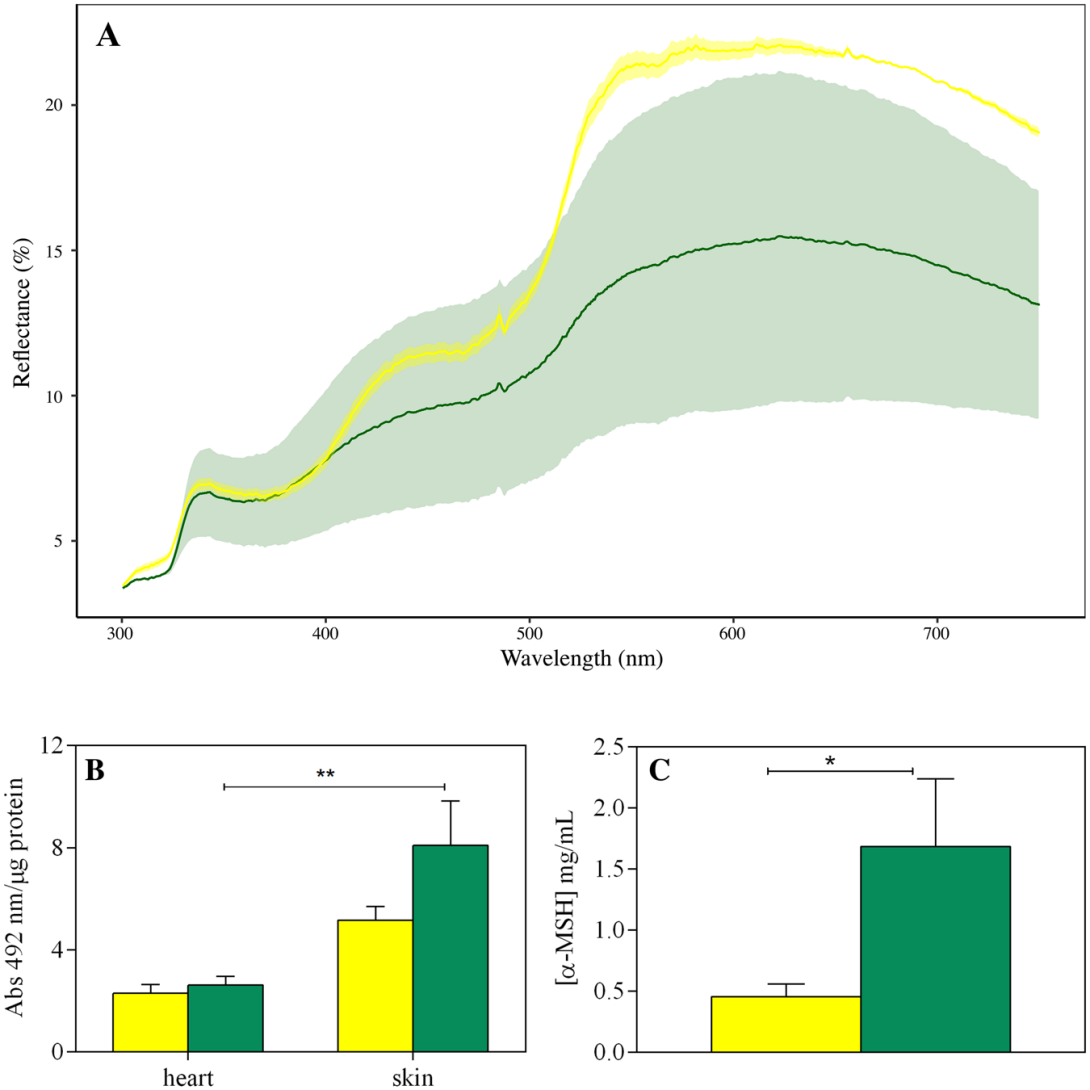
Physiological status. (**A**) Wall Gecko adjusts their dorsal colour in response to changes in environmental conditions. Increasing of reflectance was inducted by putting the animals in white box, meanwhile decreasing was observed in animals put in black box. Bold green and yellow line indicates the mean reflectance spectra (%) of the diurnal/dark and nocturnal/pale geckos respectively; the shaded areas indicate the lower reflectance measure on geckos put in black box, and the higher reflectance spectra measured on geckos put in the white box. We observed a significant variation of the skin in diurnal animals collected on olive tree (green line, N = 14), and a slightly colour variation in animals collected during the night on wall (yellow line, N = 15). Two-way ANOVA test, t = 15.149, p < 0.0001, df = 26. (**B**) Melanin assay in skin and heart and (**C**) MSH concentration in dark/diurnal (green) and pale/nocturnal (yellow) geckos.

The geckos are able to change their skin colour according to the substrate, as we previously demonstrated^29,30^. As shown in Fig. 5A, diurnal/dark geckos’ skin reflectance shows a wider variability than nocturnal/pale ones (two-way ANOVA test, t = 15.149, p < 0.0001, df = 26, see also Tables S3 and S4). Thus, diurnal/dark geckos modulate their shade of colour better than nocturnal/pale geckos, due to the amount of melanin they have in their skin.

Furthermore, the ability to change colour depends on physiological conditions^29,30^, indeed the amount of melanin is related to the levels of α-MSH plasmatic hormone. Accordingly, the determination of melanin content in the two morphotypes of geckos showed that the skin of pale geckos had lower levels of melanin than dark ones, and this is directly correlated to the lower α-MSH concentration (Fig. 5B,C; p < 0.05 unpaired t-test with Welch’s correction; df = 8). These physiological traits could determine one or other morphotype.

### Predation towards geckos

**Figure 6:**
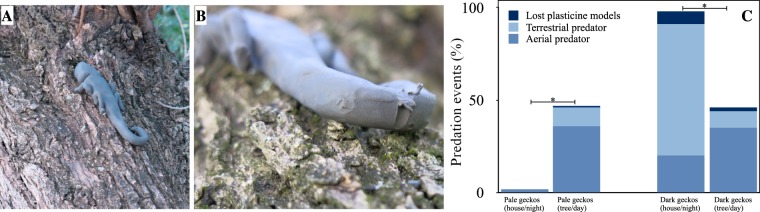
Predator attack assay. (**A**) Model of a dark gecko on the bark of an olive tree. (**B**) Predation marks on the tail of plasticine gecko. Two models for each tree/wall were placed in approximately the same position where a real gecko was previously observed (N = 40). (**C**) Predation in percentage of attacks, on pale and dark gecko models on the wall and on the tree. In parenthesis, the type of substrate on which we put the plasticine model for 24 hours. Colour indicate the Statistical analysis (chi-square test of independence) confirmed the different predation pressure observed in the two morphs on their own favorite substrate *vs* the other substrate (* chi-square P value < 0.05).

We hypothesized that predation could be significant in driving environmental background matching. In the study area, potential predators of the gecko are *Strix aluco*, *Athene noctua*, *Falco tinnunculus, Lanius collurio, Corvus corone*, and *Rattus sp*. To test this hypothesis, we used plasticine models of geckos to measure the frequency of predation events (Fig. 6)^84^. As shown in Fig. 6C, when pale models were put on walls of houses during the night, low predation events were observed, whereas when they were put on the tree trunks, during the day, about 50% showed signs of predation (chi square test p < 0.05), mainly due to aerial predators.

Dark plasticine models, put on walls of houses during the night, showed 100% of predation events with a high percentage of attacks by terrestrial predators, yet when they were put on the tree trunks during the day, the predation events decreased significantly to less than half (chi square test p < 0.05) and this was attributable to aerial predators for the most.

### Genetic characterization and population structure

We sequenced whole mitogenome from 20 geckos, to place the studied polymorphic populations into the phylogeographic context of geckos from around Italy and the Mediterranean basin. mtDNA sequences were obtained with a coverage of 14.4 ± 11.3 (mean ± std.dev; minimum = 2; maximum = 83). Phylogenetic analysis (Fig. 2), taking into account our gecko samples and sequences from external Palearctic *Tarentola* geckos, confirms that both pale and dark populations belong to the same nominal species and lineage, and all haplotypes belong to the European clade.

In order to understand the habitat-association and reproductive isolation at neutral markers, we performed population genetic analysis based on information derived from microsatellite polymorphism (see table of microsatellite in Tables S6 and S7). No evidence of linkage disequilibrium was found among 8 selected polymorphic microsatellite loci (assessed by performing correlation value permutation according to Weir^85^).

The GENELAND analysis indicated that the most likely value of *K* describing populations’ spatial clusterization was 1 (Fig. S2). GENELAND incorporates geographic coordinates of the samples into the prior parameters of the estimation procedure and *K* = 1, produced by GENELAND, is in accordance with the sympatric condition of our assumed populations.

**Figure 7:**
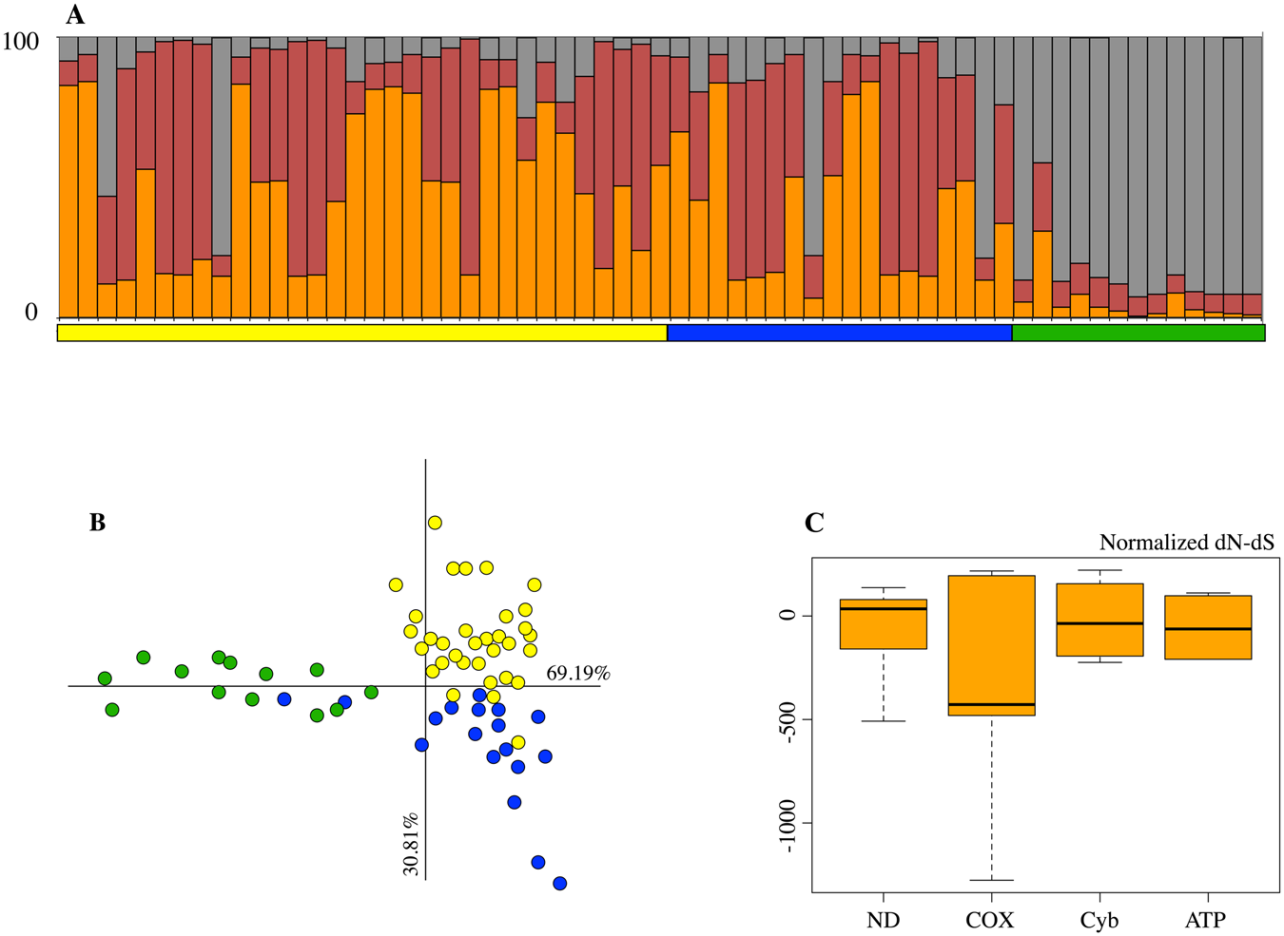
Population structure and selection. Population structure analysis of 8 microsatellite loci, (**A**) according to Structure considering an admixture model (K = 3); (**B**) according to Factorial Correspondence Analysis (FCA). It is evident a consistent gene flow for neutral markers Fst = 0.08. (**C**) Box-plot of normalized nonsynonymous (*dN*) *-* synonymous (*dS*) substitutions in diurnal and nocturnal geckos on the CDS of mtDNA, test for positive selection is not significant, *p* > 0.05. Dark/diurnal geckos (green); diurnal geckos collected on substrates different by olive tree (blue); pale/nocturnal geckos (yellow).

Instead, STRUCTURE, that does not use geographical data, combines microsatellite genotype samples in three populations^71^. Bayesian likelihood analysis, showing that *K* = 3 (Ln_P(D) = −1465.1; α = 0.0639 and ΔK = 2.0229), strongly supports the hypothesis that population is structured in three clusters (Table S7 and Fig. 7A). From the three putative populations, only diurnal/dark/trunk geckos are well characterized, whereas nocturnal/pale/wall geckos appear mixed with intermediate geckos (diurnal/no-trunk).

Factorial Correspondence Analysis (FCA) was used to generate the two components, FC1 and FC2 (x and y axes, respectively), that accounted for 69.19% and 30.81% of the total variance, respectively (Fig. 7B). Genotypes of diurnal/dark geckos, collected from olive trees, differed sharply from those of nocturnal/pale geckos collected from walls of houses. Unlike these two populations, the genotypes of intermediate geckos are arranged in a well-defined area identified by the two main components. They are aligned with the pale geckos along the first axis, and aligned with the dark geckos along the second axis.

Neutral microsatellite markers revealed a fixation index *Fst* = 0.08, p < 0.05 between diurnal/trunk and nocturnal/wall geckos; the value of index decreases between pale and intermediate populations (*Fst* = 0.03, p < 0.05), which are only separated temporally, and increases between dark and intermediate populations (*Fst* = 0.09, p < 0.05).

### Mitogenome variability and selection

Using sequences of mtDNA coding region (11,266 bp), we found that the morphs were well structured genetically, with a significant value of *Fst* = 0.38 (p < 0.05).

The sequencing of the complete mitochondrial genome showed that nocturnal geckos have lower levels of variability than diurnal ones both in nucleotide and haplotype diversity (Table S8).

The Tajima D test performed on the whole metapopulation (nocturnal and diurnal together) returned a negative value of D = −0.1292, suggesting a disruptive selection or sharp reduction in the size of population. Calculating the value of Tajima D for nocturnal and diurnal populations separately, only nocturnal geckos showed the positive value of Tajima D = 2.0449, suggesting the action of balancing selection (Table S8).

We calculated the relative frequencies of dN and dS on mitogenome sequences (Table S9).

The normalized dN-dS is a useful measure of the strength and mode of positive selection acting on protein-coding genes. Normalized dN-dS of the whole population shows close to zero or negative modal value for all coding genes (Fig. 7C), suggesting a negative selection in both populations.

Performing the permutation test^60^, the median level of dN/dS differs significantly between diurnal and nocturnal geckos (N = 7115; 100,000 permutation, dN/dS: p = 0.00091), which suggests a differential selection pressure on coding genes between two populations.

The Z-test of selection was only statistically significant for diurnal population, proving that this population differs from neutrality and is under purifying selection (Table S10).

## Discussion

Evolutionary processes acting within populations to maintain morphological polymorphisms are of particular interest as they could contribute to speciation^74^. Body and pattern colorations are morphological traits undergoing strong selection in animal species, ranging from insects to vertebrates^86^. The Wall Gecko shows heterogeneous colour pattern, which may vary among individuals, depending on the time of day and on the habitat segregation^30^ (Fig. S3). Although some zoologists detected this morphological polymorphism in sympatric populations elsewhere around the Mediterranean range^16–27^, none of them has explained this in an evolutionary context.

Here, we provide strong evidence supporting the hypothesis that the Wall Gecko is experiencing a divergence between two sympatric populations (dark and pale geckos), each adapted to a different habitat (tree trunk and wall) and temporal niches (diurnal and nocturnal).

It should be noted however that morphological variation is more complex than just nocturnal/pale *vs* diurnal/dark geckos. As a matter of fact, from visual census and reflectance analysis, we distinguished three morphotypes: pale, dark and intermediate (although the latter should not be considered a homogeneous group, but a group of individuals with a clinal variation).

In our study, the nocturnal/pale/wall and diurnal/dark/trunk geckos could be considered the extremes of an ecological cline of morphological variation, on which divergent selection may be acting. In our hypothesis the ecological segregation, which we are witnessing between the nocturnal and diurnal geckos, can lead to a speciation episode^87^.

Neutral microsatellite markers reveal that only the diurnal/dark/trunk population forms a genetically distinct group. The temporal niche (night/day) is probably less crucial in separating the populations than the spatial one (trunk/wall). This strengthens the explanation that predation is the selection force keeping the morphs in their own niche (cryptism is adaptive). Indeed, the divergence of these two sympatric populations is driven by adaptation to background matching and is associated with patterns in diurnal/nocturnal activity.

Segregation in the two spatially differentiated niches, associated with the split in circadian rhythm, may reduce gene flow and facilitate disruptive natural selection.

The *disruptive selection* happens when individuals from different populations experience multiple microhabitats, as in the case of these geckos, for which spatial and temporal heterogeneous habitats represent the main variation^88^. Indeed, light (wall during the night) and dark (trunk during the day) substrates are selected by geckos with cryptic colours as an anti-predatory strategy^89^.

Our observations can find interpretative cues from a gene flow analysis and differential variation of the mitogenome, which suggest distinct selective pressures on the two populations of geckos.

In other species, such as the classic case of the cichlids from Victoria Lake^90,91^, environmental variation is derived by a sensory recognition system, which adapts primarily to depth mediated-gradients. In our case, the main factor for the environmental variation is the time scale, with day and night as selective extremes, to which location in different substrates (tree trunks or walls) is added.

The results of the marking-release and observation experiment clearly indicate that temporally segregated geckos are not prone to changing their niche, most likely because they are conditioned by predation. Indeed the analysis of predator attacks on plasticine models of pale and dark geckos seems to suggest that selection against pale geckos, during the day, is determined by attacks from birds hunting by sight, because of the lack camouflage. On the other hand selection against dark geckos is predominantly determined by “terrestrial” predators - we observed recognisable marks of rats teeth, these animals being present during the night along with a few nocturnal predatory birds. This data reinforces the idea that divergence of gecko morphs happens in sympatry.

Some studies support the hypothesis that predators search for the most common prey types which are thus more likely to be eaten than rare morphs^92,93^. Diurnal dark geckos seem to be less frequent than nocturnal pale ones; in agreement with *frequency dependent natural selection*, these are spared and thus polymorphism is maintained in the population, the phenomenon known as *apostatic selection*^92,94^. This colour polymorphism in the Wall Gecko must be considered in the light of its ability to undergo physiological colour change, previously assessed in this species^28–30^. This ability is more evident in diurnal dark geckos than nocturnal pale ones, because it depends on the amount of melanin in the skin, although, the diurnal dark geckos cannot become as pale as the nocturnal ones and *vice versa*. The greater camouflage ability of diurnal geckos, combined with the spatial segregation on different substrates available during the day, allows them to avoid the predatory pressure better than pale geckos living on the only substrate on which it is camouflaged (the wall). In our hypothesis, the latter is affected by *balancing selection*. Combining the effect of balancing selection on nocturnal geckos and disruptive selection between two sympatric populations, could lead to speciation^95^ but may reflect a stable equilibrium between divergent selection and gene flow^95,96^.

Even when separation between incipient species seems to be the most obvious fate, the role of reproductive isolation cannot be assumed automatically^97^. As shown in the example of the Walking Sticks^98,99^ evolution of reproductive isolation depending on gene flow remains debatable. However, some theoretical analyses increasingly support the plausibility of reinforcement, in which evolution of reproductive isolation is promoted by mating between incipient species^100^. In our study, the widespread occurrence of two morphs (dark and pale geckos in sympatry, each adapted to a different microhabitat) may suggest also that we are witnessing an ancient polymorphism with leaky reproductive isolation that rarely (if ever) progresses beyond the incipient speciation stage as observed in other species^95^.

Starting from field observations following a bottom-up approach and using physiological and genomic data, we provide evidence, which sheds new light on sympatric speciation, a controversial process never previously described for this species.

## Supplementary information

S1

## Data Availability

The data that support the findings of this study are deposited in GenBank under accession numbers from MK275668 to MK275687.
