# Author Correction: Selection for background matching drives sympatric speciation in Wall Gecko

**DOI:** 10.1038/s41598-019-53470-1

**Published:** 2019-11-08

**Authors:** Domenico Fulgione, Maria Buglione, Daniela Rippa, Martina Trapanese, Simona Petrelli, Daria Maria Monti, Massimo Aria, Rita Del Giudice, Valeria Maselli

**Affiliations:** 1 Department of Biology, University of Naples Federico II, Via Cupa Nuova Cinthia 26, 80126 Naples, Italy; 2 Department of Chemical Sciences, University of Naples Federico II, Via Cupa Nuova Cinthia 26, 80126 Naples, Italy; 3 Department of Economics and Statistics, University of Naples Federico II, Via Cupa Nuova Cinthia 26, 80126 Naples, Italy

Correction to: *Scientific Reports* 10.1038/s41598-018-37587-3, published online 04 February 2019

The original version of this Article was published with an incorrect version of the Supplementary Information file. This has been corrected in the HTML version of the Article; the PDF version was correct at time of publication.

