# Supplementary material for: Selection for background matching drives sympatric speciation in Wall Gecko
Source: Sci Rep. 2019 Feb 4;9:1288. doi: 10.1038/s41598-018-37587-3 (PMC6361904; doi:10.1038/s41598-018-37587-3)
Supplement: Supplementary file 1 — S1 [file 41598_2018_37587_MOESM1_ESM.docx]

**Selection for background matching drives sympatric speciation in Wall Gecko**

Domenico Fulgione^1*^, Maria Buglione^1^, Daniela Rippa^1^, Martina Trapanese^1^, Simona Petrelli^1^, Daria Maria Monti^2^, Massimo Aria^3^, Rita Del Giudice^2^ and Valeria Maselli^1^

^1^Department of Biology, University of Naples Federico II, Via Cupa Nuova Cinthia 21, 80126 Naples, Italy

^2^Department of Chemical Sciences, University of Naples Federico II, Via Cupa Nuova Cinthia 21, 80126 Naples, Italy

^3^Department of Economics and Statistics, University of Naples Federico II, Via Cupa Nuova Cinthia 21, 80126 Naples, Italy

*Correspondence to: Domenico Fulgione, [fulgione@unina.it](mailto:fulgione@unina.it)

**Supplementary information**

**Table S1. Conditional distributions.** The conditional distributions show that dark geckos are preferably associated to the tree (86.2% of the geckos found on the trees are dark), whereas pale ones are preferentially associated to the walls (73.4% of the geckos found on the walls are pale). D, I and P represent dark, intermediate and pale geckos, respectively.

|  | | **Location** | | | **Total** |
| --- | --- | --- | --- | --- | --- |
|  |  | **others** | **tree** | **wall** |  |
| **Colour** | **D** | 0.0% | 86.2% | 8.3% | 27.7% |
|  | **I** | 75.0% | 13.8% | 18.3% | 18.2% |
|  | **P** | 25.0% | 0.0% | 73.4% | 54.1% |
| **Total** | | 100.0% | 100.0% | 100.0% | 100.0% |

**Table S2. Association between location and colour.** Statistical analysis testing association between location (wall, tree) and colour (pale, intermediate and dark). Based on 10,000 sampled tables with starting seed 957,002,199. The association between the location and colour variables is positive for all the performed tests. By elaborating a causal analysis with the Tau index of Goodman and Kruskal, we have measured the causal relationship between the two variables. Specifically, considering the coluor as a causality and the location as an effect (dependent) the index is 0.555 (the maximum is 1), and is statistical significant (P < 0.001).

|  | **Value** | **df** | **Asymp. Sig. (2-sided)** | **Monte Carlo Sig. (2-sided)** | | |
| --- | --- | --- | --- | --- | --- | --- |
|  |  |  |  | **Sig.** | **99% Confidence Interval** | |
|  |  |  |  |  | **Lower Bound** | **Upper Bound** |
| **Pearson Chi-Square** | 147.079 | 4 | 0.000 | 0.000^b^ | 0.000 | 0.000 |
| **Likelihood Ratio** | 158.305 | 4 | 0.000 | 0.000^b^ | 0.000 | 0.000 |
| **Fisher's Exact Test** | 151.898 |  |  | 0.000^b^ | 0.000 | 0.000 |
| **N of Valid Cases** | 231 |  |  |  |  |  |

**Table S3. Analysis of variance of reflectance, computed against model Y=Mean (Y)**

| **Source** | **DF** | **Sum of squares** | **Mean squares** | **F** | **Pr > F** |
| --- | --- | --- | --- | --- | --- |
| Model | 3 | 70628719.882 | 23542906.627 | 2746.439 | < 0.0001 |
| Error | 56 | 480040.782 | 8572.157 |  |  |
| Corrected Total | 59 | 71108760.664 |  |  |  |

**Table S4. Model parameters of reflectance.**

| **Source** | **Value** | **Standard error** | **t** | **Pr > \|t\|** | **Lower bound (95%)** | **Upper bound (95%)** |
| --- | --- | --- | --- | --- | --- | --- |
| Intercept | 2553.429 | 23.906 | 106.813 | **< 0,0001** | 2505.540 | 2601.317 |
| Day *vs* Night gekos | -2233.371 | 33.808 | -66.061 | **< 0,0001** | -2301.096 | -2165.647 |
| White *vs* black box | 512.138 | 33.808 | 15.149 | **< 0,0001** | 444.413 | 579.863 |
| Type*black box | 376.432 | 47.811 | 7.873 | **< 0,0001** | 280.655 | 472.209 |

**Table S5. Primer sequences and descriptions of the eight microsatellites.**

| **Locus (accession number)** | **Primer sequence (5' - 3')** | **Repeat array in clone** | **Clone size range (bp)** | **# alleles** |
| --- | --- | --- | --- | --- |
|  |  |  |  |  |
| Tb35 (HM212432) | AATTATCCTGTTTGTGATTTTGG TAGAGACGTCAAATGCTGCGTC | (GAAA)_23_ | 375 - 459 | 9 |
| Tb8 (HM212431) | ACCACATTGGTAACCTTTACTTGCC GATAATCTGTTTTGAAGTTTTGGAAC | (GAAA)_22_ | 210 - 280 | 9 |
| Tb71  (HM212427) | CTGGACTTGTACTTTATTCTGATGC  GTAGTTACCATGGCTAGTAGCC | (GAAA)_23_ | 324 - 395 | 9 |
| Tb192 (HM212429) | GATGGAAAATCCAGTTCAAGGAGG GCACATTGAGGAAAAGCATCTAC | (GAAA)_17_ | 356 - 441 | 12 |
| Tb213  (HM212434) | GATCCTCACATAATAAATGC TCTATCTTGAGGCACCACATG | (GAAA)_21_ | 459 - 540 | 7 |
| Tb240  (HM212430) | ATCACATATCATGGCCTAGGG ATGCCCATAGGAATATTGTAGCC | (GAAA)_6_(GA)_5_ (GAAA)_6_ | 504 - 577 | 8 |
| Mt6 (KC470204) | TCCACTGAGGTCCTGTTCC  TGGTTGCAACAGACCAACT | (AC)_11_ | 157-179 | 3 |
| Mt27 (KC489794) | GAGAGCCTGGTTCAAAT  ACTCCACTGATTCCCCTCAA | (GA)_9_ | 153-185 | 2 |

**Table S6**. Microsatellite diversity in metapopulation. He, mean expected heterozygosity; He SD, standard deviation of expected heterozygosity; Ho, mean observed heterozygosity; Ho SD, standard deviation of observed heterozygosity.

|  | **He** | **He SD** | **Ho** | **Ho SD** |
| --- | --- | --- | --- | --- |
| **Pale** | 0.6628 | 0.0936 | 0.6503 | 0.1745 |
| **Intermediate** | 0.6034 | 0.2335 | 0.5945 | 0.3154 |
| **Dark** | 0.5158 | 0.2036 | 0.4537 | 0.2528 |

**Table S7**. Bayesian clustering analysis implemented in Structure, on microsatellite data.

|  | **Ln_P(D)** | **α** | **ΔK** |
| --- | --- | --- | --- |
| ***K = 1*** | -1627.5 | - | - |
| ***K = 2*** | -1542.9 | 0.0897 | 0.0585 |
| ***K = 3*** | -1465.1 | 0.0639 | 2.0229 |
| ***K = 4*** | -1413.8 | 0.0491 | 0.2175 |
| ***K = 5*** | -1397.3 | 0.0453 | 0.0777 |

**Table S8**. Genetic variability and Tajima D test for whole mitogenome (mtDNA) and coding genes separately of Wall Gecko.

| **Region** | **Length** | **π** | **Hd** | **VarHd** | **TajimaD** |
| --- | --- | --- | --- | --- | --- |
| **whole mtDNA nocturnal geckos** | 16605 | 0.00016 | 0.618 | 0.01078 | 2.04492 |
| **whole mtDNA diurnal geckos** | 16605 | 0.00139 | 0.722 | 0.02535 | -0.48280 |
| **coding genes nocturnal geckos** | 11266 | 0.00014 | 0.509 | 0.01015 | 1.68091 |
| **coding genes diurnal geckos** | 11265 | 0.00121 | 0.722 | 0.02535 | -088757 |

**Table S9**. Analysis of the frequency of non-synonymous (*dN*) and synonymous (*dS*) substitutions in diurnal *vs* nocturnal geckos’ populations.

|  | **%dS ± s.e.** | | | **%dN ± s.e.** | | | | **dN/dS** |
| --- | --- | --- | --- | --- | --- | --- | --- | --- |
| **ND** | 0.2340 | **±** | 0.0034 | | 0.1040 | **±** | 0.0012 | 0.44 |
| **COX** | 0.0544 | **±** | 0.0032 | | 0.0086 | **±** | 0.0006 | 0.16 |
| **ATP** | 0.0200 | **±** | 0.0051 | | 0.0094 | **±** | 0.0024 | 0.47 |
| **cytb** | 0.0173 | **±** | 0.0033 | | 0.0139 | **±** | 0.0029 | 0.81 |
| **TOTAL** | 0.3467 | **±** | 0.0025 | | 0.1644 | **±** | 0.0010 | **0.47** |

**Table S10**. Z-tests value for diurnal and nocturnal populations.

|  |  | **Diurnal** | **p-value** | **Nocturnal** | **p-value** |
| --- | --- | --- | --- | --- | --- |
| Z-test | **Positive selection** | -2.139 | 1 | -0.316 | 1 |
|  | **Neutral** | -2.160 | 0.033 | -0.320 | 0.749 |
|  | **Purifying selection** | 2.102 | 0.019 | 0.315 | 0.377 |

**
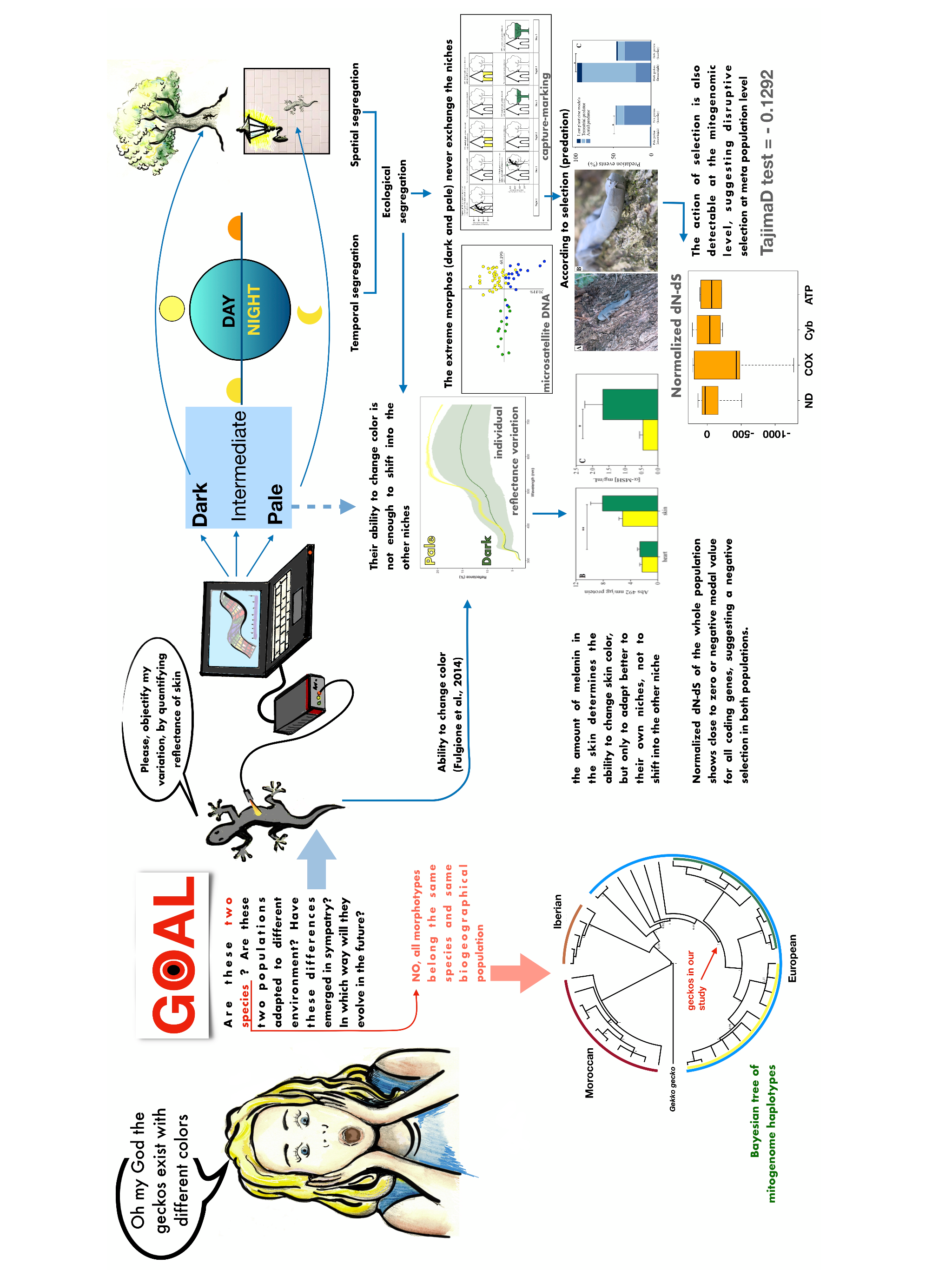
**

**Fig. S1. Graphical Abstract**


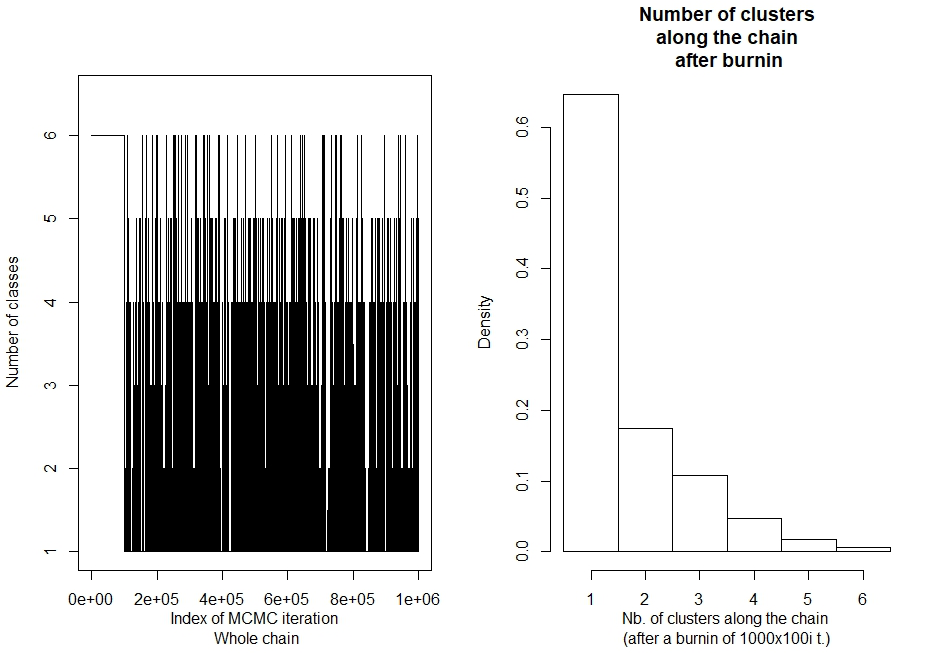


**Figure S2:** Number of populations simulated from the posterior distribution with GENELAND package in R. The run displays a clear mode at *K* = 1, which is the maximum *a posteriori* estimate of *K*.


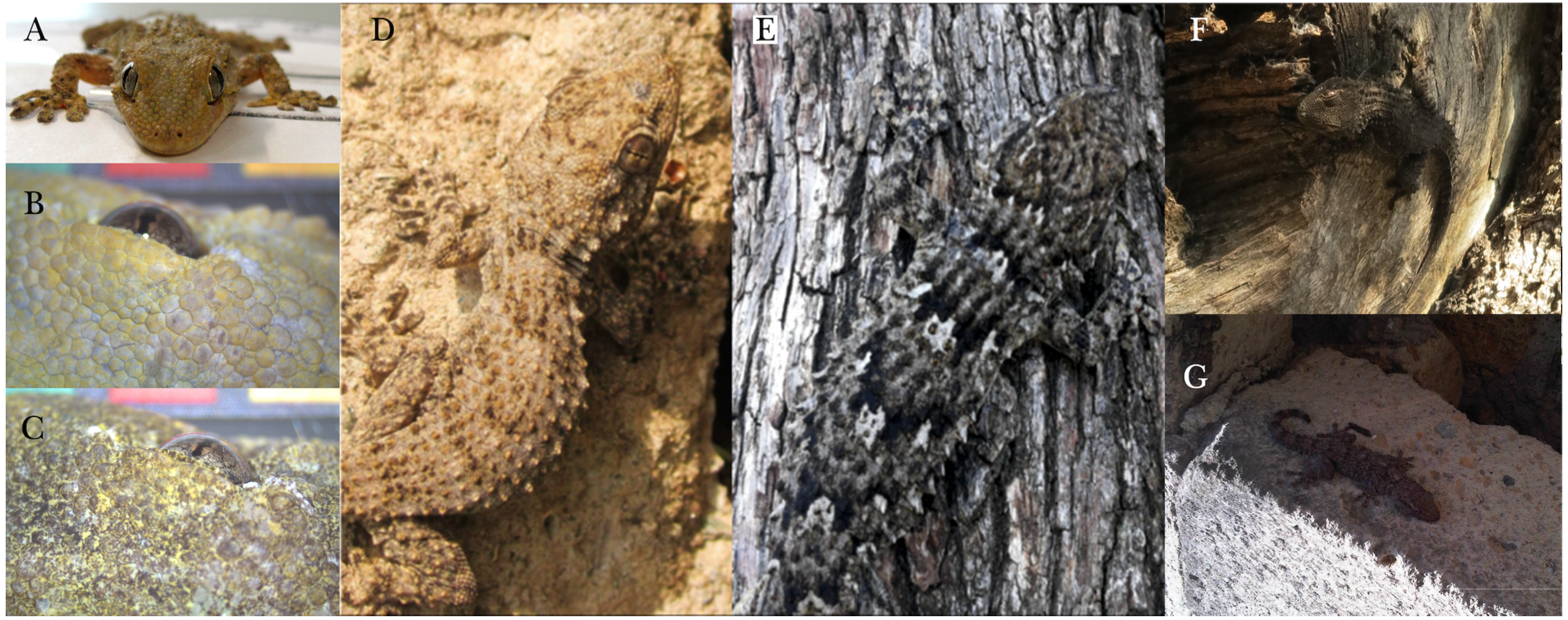


**Fig. S3. Wall Geckos heterogeneous colour pattern and segregation**. The gecko (A) is able to change color according to the substrate, darkening and clearing, condensing (B) or dispersing (C) the available melanin in the skin, within about one hour. Geckos with less melanin live on light substrates during the night (D), while those with greater amounts of melanin choose dark substrates (E). The latter prefer tree trunks because they are cryptic during the day (F), but more rarely they are found on walls during the day, though always in the shade and in less detectable condition (G).
